# Emerging Socioeconomic Disparities in COVID-19 Vaccine Second-Dose Completion Rates in the United States

**DOI:** 10.3390/vaccines10010121

**Published:** 2022-01-14

**Authors:** Autumn Gertz, Benjamin Rader, Kara Sewalk, John S. Brownstein

**Affiliations:** 1 Computational Epidemiology Laboratory, Boston Children’s Hospital, Boston, MA 02115, USA; autumn.gertz@childrens.harvard.edu (A.G.); benjamin.rader@childrens.harvard.edu (B.R.); kara.sewalk@childrens.harvard.edu (K.S.); 2 Department of Epidemiology, Boston University School of Public Health, Boston, MA 02118, USA; 3 Department of Pediatrics, Harvard Medical School, Boston, MA 02115, USA

**Keywords:** COVID-19, vaccination, inequity, multi-dose vaccines, health disparities

## Abstract

Although COVID-19 vaccination plans acknowledge a need for equity, disparities in two-dose vaccine initiation have been observed in the United States. We aim to assess if disparity patterns are emerging in COVID-19 vaccination completion. We gathered (*n* = 843,985) responses between February and November 2021 from a web survey. Individuals self-reported demographics and COVID-19 vaccination status. Dose initiation and completion rates were calculated incorporating survey weights. A multi-variate logistic regression assessed the association between income and completing vaccination, accounting for other demographics. Overall, 57.4% initiated COVID-19 vaccination, with 84.5% completing vaccination. Initiation varied by income, and we observed disparities in completion by occupation, race, age, and insurance. Accounting for demographics, higher incomes are more likely to complete vaccination than lower incomes. We observe disparities in completion across annual income. Differences in COVID-19 vaccination completion may lead to two tiers of protection in the population, with certain sub-groups being better protected from future infection.

## 1. Introduction

Equitable distribution of the COVID-19 vaccine in the United States (USA) is necessary to prevent exacerbation of health disparities [1]. Yet, early evidence suggests that lower-income, non-White, and older individuals have disproportionately lower vaccine rates [2], a trend mirroring the stark inequalities observed in other adult immunizations [3]. Disparities across race, ethnicity, and age have been previously reported for the administration of the influenza, pneumococcal, and hepatitis B immunizations [3]. The observation of these differences has been partially attributed to the complex interplay of healthcare access, literacy, and attitudes [3].

Early reports from the USA Centers for Disease Control and Prevention (CDC) have indicated that COVID-19 vaccination coverage has been following a similar trend. Disparities in vaccination have been observed across population density, age, and sex [4,5]. While COVID-19 vaccination prioritization plans have acknowledged a need for equity, the primary focus has targeted reducing deaths and severe illness [6]. Empirical guidelines for the currently available mRNA formulas of COVID-19 vaccines recommend two-doses for “full vaccination”. The requirement for a second dose has introduced an additional axis for disparities to grow [7,8]. Previous analyses of multi-dose vaccines suggest that minorities and individuals with poor insurance coverage were less likely to complete vaccine regimens irrespective of initiation rates [6].

Lower rates of COVID-19 vaccine initiation have been observed among groups with social vulnerabilities [9]. Assessment of the first phase of COVID-19 vaccinations in December 2020 and January 2021 found a higher proportion of females, adults over fifty, and non-Hispanic Whites recipients [10]. Among those who were prioritized initially, overall completion rates are high for COVID-19 vaccinations [9]. The COVID-19 vaccine distribution was initially targeted at highly vulnerable groups including long-term care residents, individuals with multiple comorbidities, and healthcare workers [11]. However, disparities in receiving the second dose within the recommended time interval were observed across race, ethnicity and age during the initial rollout of the vaccine [12].

As of April 2021, all USA adults were eligible to receive the COVID-19 vaccine, regardless of age, risk, professional setting, or state-specific prioritization guidelines [13]. In addition to widespread eligibility, widespread availability of vaccine appointments also expanded, with clinics established at a variety of locations including healthcare facilities, retail pharmacies, and mass-vaccination sites [14]. Understanding disparities that developed in initial COVID-19 vaccine completion will help to inform equitable administration of additional doses. This study aims to assess if the disparity patterns observed in other adult immunizations and COVID-19 vaccine initiation are emerging in COVID-19 vaccination completion during nine months of widespread vaccine administration.

## 2. Materials and Methods

We gathered (*n* = 843,985) responses between 8 February and 14 November 2021 from a previously validated [15] web survey administered by OutbreaksNearMe.org on Momentive.ai (previously SurveyMonkey, 850,586 fielded, 99.2% completion rate). National general population weights for gender, age, race, education, geography, and profession were applied to reflect USA Census targets, enabling better representation of the USA population. An additional weighting parameter for political identification was included. Survey weights are generated daily during weekdays and once after weekends. Individuals self-reported personal demographics, pandemic related behaviors, and COVID-19 vaccination status. Specifically, each respondent was asked a two-question series: 1. “Did you get the COVID-19 vaccine?” and if yes, 2. “Have you received all required doses?” To accommodate booster dose availability, the second question was changed (on 23 August 2021) to: “Have you received all required doses (excluding booster doses)?” Both questions could be answered Yes/No, and individuals were, respectively, categorized as not vaccinated, partially vaccinated, or fully vaccinated.

Dose initiation rates were calculated as number who received at least one dose over total population, and dose completion rates were calculated as number of completed vaccinations (either two-doses of mRNA formula or one dose of viral vector vaccine) over the number who received at least one dose. The number of completed vaccinations was determined by a “yes” response to “Have you received all required doses?” Dose initiation and completion rate calculations incorporated survey weights (targeting USA census defined population demographics), are presented with margin-of-error intervals accounting for design effect, and are compared across demographics. An additional question added to the survey on 8 March 2021 allowed us to do a sub-analysis of completion rates among only those (*n* = 485,714) who reported receiving one of the two available mRNA vaccine formulas.

A multi-variate logistic regression model was fit to assess the association between annual household income and the likelihood of completing COVID-19 vaccination among those who had initiated vaccination, while accounting for other demographics (age, race, health insurance coverage, essential worker status, professional industry, and political party). Professional industries were categorized as healthcare, education, government, other, and missing. Odds ratios and 95% confidence intervals were calculated from the multi-variate regression and are presented. Dose initiation rates, dose completion rates, and multi-variate logistic regression were also stratified by healthcare worker status. Analyses were conducted in R version 4.0.4. This study was approved by Boston Children’s Hospital Institutional Review Board and received a waiver of informed consent.

## 3. Results

### 3.1. Vaccine Initiation and Completion

Among all respondents over the nine-month study period, incorporating survey weights, 57.4% (*n* = 565,144) indicated initiating the COVID-19 vaccine, and 48.5% (*n* = 488,839) indicated being fully vaccinated. Of those who had received at least one dose, 84.5% had completed their vaccination. Vaccination initiation varied substantially by income with higher household incomes associated with higher initiation rates (Table 1). Among individuals in the highest annual income bracket (>$150,000), 69.4% had initiated COVID-19 vaccination. Conversely, in the lowest income bracket (less than $30,000 a year), only 43.6% had initiated vaccination. Differences in vaccination completion across income were less pronounced, with the highest income bracket slightly higher (87.6%) than the lowest (80.1%) (Table 1). When analyzing rate differences by occupation, race, age, and insurance type, we find larger disparities. Healthcare workers had the highest dose initiation (73.3%) and completion (91.4%) rates (Table 1). Self-reported essential workers had a low dose initiation (56.1%), but high dose completion (85.6%) rate (Table 1). Individuals 65 and older had a higher dose initiation rate (82.1%), and higher completion rate (87.3%) compared to other age groups. Among uninsured individuals, dose initiation (34.3%) and dose completion (77.4%) rates were lower than all insured groups. Dose initiation varied more by race than dose completion (Table 1). The highest initiation rate was among Asians (67.7%) followed by Whites (59.9%). Both Blacks (49.5%) and Hispanics (52.3%) had lower initiation rates. Across race, dose completion ranged from 81.4% to 85.7%. A similar pattern was observed by political party, with varied initiation rates but comparable completion rates (Table 1).

When stratified by healthcare worker status, similar trends in dose initiation and completion across age, race, political party, and income were observed in both strata, the information is in Supplementary Materials (Table S1). Healthcare workers who identified as essential workers had higher initiation (74.3%) and completion (92.0%) rates while non-essential non-healthcare workers had higher initiation (60.5%) and completion rates (84.3%). Non-healthcare workers with Medicare had the highest initiation rate (67.5%), while healthcare workers insured through their employer had the highest initiation rate (78.2%). Despite differences in initiation across healthcare coverage, healthcare and non-healthcare workers had similar trends in dose completion rates. Both healthcare and non-healthcare workers without insurance had the lowest dose initiation and dose completion rates (Table S1).

### 3.2. Likelihood to Complete Vaccination by Income

**Figure 1:**
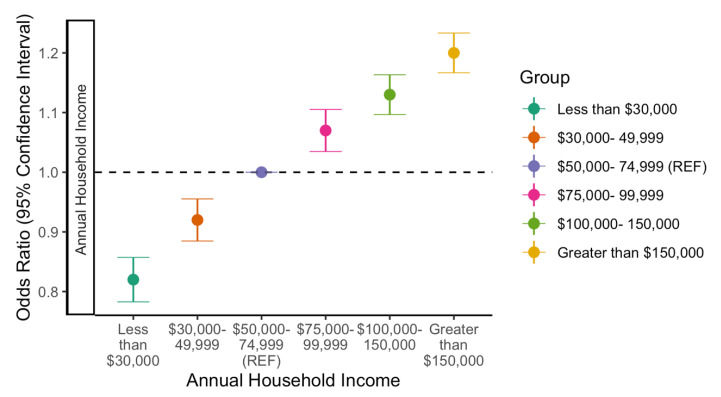
Likelihood to complete COVID-19 vaccination by annuals household income adjusted for age, race, health insurance, professional industry, essential worker status, and political party.

More profound trends in disparities across annual household income were observed in likelihood to complete COVID-19 vaccine when adjusting for other demographic covariates (as estimated by adjusted odds ratio (AOR) statistics from logistic regression analyses). In general, we see a linear pattern by which each higher income bracket is progressively more likely to have completed COVID-19 vaccination (Figure 1). For example, those with an annual household income of less than $30,000 a year had 0.82 the odds (95% CI: 0.78 to 0.86) of successful vaccine completion compared to those making $50,000–74,999 a year (Figure 1). Conversely, those in the highest income bracket (>$150,000) had 1.20 (95% CI: 1.17 to 1.23) the odds of vaccine completion compared to the reference group, even when adjusted for profession and other demographic covariates (Figure 1). The regression displays disparities in likelihood to complete COVID-19 vaccination across income compared to medial income group, while weighted completion rates showed less pronounced differences. Full regression results are located in Table S2.

A similar linear pattern across annual household income was observed among healthcare workers and non-healthcare workers regarding likelihood to complete COVID-19 vaccination. Healthcare workers making less than $30,000 a year had 0.72 the odds of completing vaccination compared to those making $50,000–74,999 annually (95% CI: 0.64 to 0.80). Compared to the same annual income among non-healthcare workers, those making less than $30,000 annually had 0.86 the odds of successfully completing vaccination (95% CI: 0.82 to 0.90). Comparable to the assessment of the entire study population, those in the highest income bracket among healthcare (AOR = 1.66, 95% CI: 1.54 to 1.78) and non-healthcare workers (AOR = 1.20, 95% CI: 1.16 to 1.24) had the highest odds of COVID-19 vaccine completion. The full regression output stratified by healthcare worker status is located in Table S3.

## 4. Discussion

Examining and mitigating emerging disparities in COVID-19 vaccine completion is crucial to ensuring long-term vaccination is equitable and successful. While a single dose of the mRNA vaccine has shown high effectiveness, especially among those who were previously infected with COVID-19 [16], receiving at least two doses remains the recommendation to maximize immunogenicity [8]. Differences in those who have completed vaccination of the available mRNA vaccines may lead to two tiers of protection in the USA population, with certain sub-groups being better protected from future infection. Our findings provide early insight into second-dose disparities emerging in COVID-19 vaccination. These findings of who is partially vaccinated and who is fully vaccinated may be further exacerbated by the optional recommendation of booster doses for all adults in the USA [8,17,18].

The initial prioritization of vaccinating healthcare workers and the elderly may explain their higher vaccine completion rate. This prioritization also potentially lends to greater completion rates in higher annual incomes, and insured groups. In addition to an early focus on vaccination, clinics within hospitals reduced access barriers for healthcare workers, and they have had multiple months to receive a second dose. Groups of healthcare workers may also be working at institutions with COVID-19 vaccine mandates which would require vaccine series completion. Conversely, individuals with a lower annual income, and non-healthcare essential workers had to locate and travel to vaccine clinics to receive doses [19], presenting additional access barriers to full dose completion. All COVID-19 vaccinations are freely available to USA residents, regardless of insurance status [20], a measure implemented to help mitigate financial barriers to vaccination. However, when adjusting for demographic covariates including professional industry and health insurance coverage, we still found large disparities across annual household income in their likelihood to have completed COVID-19 vaccination. Furthermore, when stratifying by healthcare worker status, disparate trends in completion across income persisted. Higher annual incomes are associated with higher dose completion rates and, when accounting for other demographics, higher likelihood to have completed COVID-19 vaccination. Completion rates across annual income were all high and comparable, but those with higher income were still observed to have the highest completion rate. The same disparities seen with COVID-19 related outcomes such as infections, hospitalizations, and deaths are persisting with COVID-19 vaccine completion rates. This suggests that in the long term, current outcome disparities may continue to widen.

Understanding the disparities associated with a multi-dose vaccination campaign, such as those observed across income, will become increasingly important as the COVID-19 vaccine administration is expanded to a pediatric population. Previous research into other multi-dose adolescent vaccinations, such as the human papilloma virus (HPV) vaccine, found significant disparities among individuals who completed all doses in the three-dose series [7,21]. Similar to results shown here, adolescents who were Black, Hispanic, and came from households with lower incomes all had lower vaccine completion rates. If the same patterns observed in adult COVID-19 and adolescent HPV multi-dose vaccination extend to adolescent and pediatric COVID-19 vaccination, we can risk further aggravation of disparate outcomes in a highly vulnerable population. Conversely, the COVID-19 pandemic may positively influence attitudes towards vaccination among vulnerable sub-groups, such as pregnant women, and lessen disparities observed in other vaccinations [22]. It will be important to monitor these disparities over time as the COVID-19 pandemic may alter long-term perceptions of vaccination among varying sub-groups.

There are multiple limitations to this study. While the dynamic nature of this survey means sub-groups who were more recently eligible to receive their first dose may not yet have had the opportunity to complete vaccination, the current results are presented with over nine months of data. Despite this lag, the observed gaps are larger than can be realistically overcome in just three-weeks of additional administration (i.e., the recommended timing between the first and second dose of the Pfizer vaccine). The majority of USA adults have had sufficient time to initiate and complete the vaccine regimen within our nine-month study period. Our data structure does not allow the full delineation of those who received a viral-vector (single-dose) or mRNA (two-dose) vaccine. The inclusion of viral vector vaccine recipients in the vaccine completion group may understate observed disparities. However, when restricting to data prior to the approval and administration of the viral-vector vaccine, similar completion trends across groups were observed (Table S4). Furthermore, a sub-analysis of those who indicated receiving an mRNA vaccine displayed similar disparate patterns in completion rates (Table S5). These results are also based on internet survey research which may be susceptible to social desirability and selection bias, especially for a politically sensitive topic such as COVID-19 vaccination. While there is uncertainty regarding the overall representativeness of survey results, the anonymous nature of our queries and the use of survey weights to adjust for demographics have resulted in overall vaccination rate estimates that are highly correlated with the USA CDC’s official estimates. This survey has also been shown to track closely with alternative estimates of COVID-19 related behavior such as mask-wearing across the geography of the USA [15]. There are other socioeconomic status factors not explored here that may interplay with the disparities we observed, or contribute to vaccine initiation and completion across groups.

Disparities in COVID-19 vaccine completion may be an early sign of what the USA will experience with the rollout of boosters. Emergence of disparate booster vaccinations may be observed among those who received a viral vector COVID-19 vaccine, where a single dose constitutes vaccine completion [8]. While for most the type of vaccine received was based on availability at a given location, individuals with potentially more barriers to vaccination may have sought out a single-dose vaccine, rather than multi-dose, to ease vaccine completion. More sociobehavioral factors such as attitudes towards vaccines, science, and politics, as well as access and susceptibility to disinformation may also contribute to differences in vaccine completion, and willingness to comply with changing recommendations [23]. These sociobehavioral factors are potentially linked to income and other demographics with observed disparities. Psychological defense mechanisms relating to the COVID-19 pandemic may also influence vaccine acceptance and compliance, as the pandemic continues to progress. [24]. Vaccine acceptance may vary by socioeconomic status and sociobehavioral dynamics, delaying vaccine initiation and potentially contributing to the disparities observed in completion. As we learn more about the role of booster doses in sustained COVID-19 immunity, information gleaned from vaccine completion coverage, such as differences across annual income, will be important in promoting equity for the subsequent phases of vaccination. Even mild disparities can have consequential public health impacts when played out on a national scale. Here, we find that higher annual incomes are associated with higher COVID-19 vaccine completion rates, suggesting that the same disparities seen with other adult immunizations, COVID-19 related outcomes, and vaccine initiation are remerging within COVID-19 vaccine completion. As COVID-19 vaccine administration continues for both initial and booster doses, completion rate changes will be important to monitor and access barriers to vaccine completion must be identified and addressed.

## Figures and Tables

**Table 1:** COVID-19 Vaccination Initiation and Completion Rates by Demographics.

Demographic	Total ^a^ No. (%)	Dose Initiation ^b^ (95% MOE) ^c^	Dose Completion ^b^ (95% MOE) ^c^
Age (years)			
18–34	151,329 (17.9)	43.1 (42.8–43.4)	81.5 (81.1–81.9)
35–44	134,326 (15.9)	49.6 (49.2–50.0)	83.8 (83.4–84.2)
45–54	167,946 (19.9)	59.4 (59.1–59.7)	85.2 (84.9–85.5)
55–64	193,074 (22.9)	69.3 (69.0–69.6)	85.7 (85.4–86.0)
≥65	185,594 (22.0)	82.1 (81.9–82.3)	87.3 (87.1–87.5)
Missing	11,716 (1.4)	Suppressed ^d^	Suppressed ^d^
Race			
White	557,068 (66.0)	59.9 (59.7–60.1)	85.7 (85.5–85.9)
Black	115,265 (13.7)	49.5 (49.0–50.0)	81.4 (80.9–81.9)
Hispanic	87,105 (10.3)	52.3 (51.8–52.8)	81.5 (81.0–82.0)
Asian	40,563 (4.8)	67.7 (67.0–68.4)	84.6 (84.0–85.2)
Other	43,984 (5.2)	46.0 (45.3–46.7)	83.5 (82.7–84.3)
Essential Worker			
Yes	379,082 (44.9)	56.1 (55.9–56.3)	85.6 (85.4–85.8)
No	200,384 (23.7)	60.9 (60.6–61.2)	84.5 (84.2–84.8)
Missing	264,519 (31.3)	52.2 (50.1–54.3)	86.3 (84.4–88.2)
Industry			
Healthcare	142,739 (16.9)	73.3 (72.9–73.7)	91.4 (91.1–91.7)
Education	66,448 (7.9)	66.8 (66.2–67.4)	83.9 (83.4–84.4)
Government	34,742 (4.1)	66.6 (65.8–67.4)	86.6 (85.9–87.3)
Other	311,313 (36.9)	53.8 (53.5–54.1)	83.9 (83.6–84.2)
Missing	288,743 (34.2)	57.2 (56.9–57.5)	83.5 (83.2–83.8)
Annual Household Income			
<30,000	156,355 (18.5)	43.6 (43.2–44.0)	80.1 (79.7–80.5)
30–49,999	117,278 (13.9)	54.8 (54.4–55.2)	83.3 (82.9–83.7)
50–74,999	133,339 (15.8)	59.4 (59.0–59.8)	84.8 (84.4–85.2)
75–99,999	110,193 (13.1)	62.6 (62.2–63.0)	85.7 (85.3–86.1)
100–150,000	136,881 (16.2)	65.7 (65.3–66.1)	86.4 (86.1–86.7)
>150,000	134,168 (15.9)	69.4 (69.0–69.8)	87.6 (87.6–87.3)
Missing	55,771 (6.6)	63.5 (62.9–64.1)	85.5 (85.0–86.0)
Health Insurance			
Plan through employer	399,956 (47.4)	60.9 (60.7–61.1)	85.7 (85.5–85.9)
Medicare	171,949 (20.4)	67.6 (67.3–67.9)	85.5 (85.2–85.8)
Self-purchased plan	91,780 (10.9)	57.4 (56.9–57.9)	84.8 (84.3–85.3)
Medicaid or Medi-Cal	57,940 (6.9)	41.0 (40.4–41.6)	79.3 (78.5–80.1)
Tricare	15,560 (1.8)	59.4 (58.2–60.6)	85.0 (83.9–86.1)
Other	49,849 (5.9)	53.9 (53.2–54.6)	82.2 (81.5–82.9)
Uninsured	45,970 (5.4)	34.3 (33.6–35.0)	77.4 (76.5–78.3)
Missing	10,981 (1.3)	48.1 (46.6–49.6)	82.5 (81.0–84.0)
Political Party			
Republican	188,701 (22.4)	53.3 (52.9–53.7)	85.0 (84.7–85.3)
Democrat	332,426 (39.4)	68.2 (68.0–68.4)	84.9 (84.7–85.1)
Independent	281,350 (33.3)	51.4 (51.1–51.7)	83.6 (83.3–83.9)
Missing	41,508 (4.9)	55.1 (54.4–55.9)	83.3 (83.1–84.5)

^a^ Crude, unweighted responses; ^b^ rates are weighted for gender, age, race, education, geography, profession, and political identification to match national general population; ^c^ margin of error (MOE; ^d^ sub-groups with insufficient numbers of responses were suppressed.

## Data Availability

Public health practitioners can request aggregate data by emailing info@outbreaksnearme.org.

## Supplementary Materials

The following supporting information can be downloaded at: https://www.mdpi.com/article/10.3390/vaccines10010121/s1, Table S1: COVID-19 Vaccination Initiation and Completion Rates by Demographics, Stratified by Healthcare Worker status; Table S2: Full Regression Output for Association between Annual Household Income and COVID-19 Vaccine Completion; Table S3: Full Regression Output for Association between Annual Household Income and COVID-19 Vaccine Completion, Stratified by Healthcare Worker Status,; Table S4: COVID-19 Vaccination Initiation and Completion Rates by Demographics, Prior to Approval and Administration of Viral-Vector Vaccine; Table S5: COVID-19 Vaccination Completion Rates by Demographics, among Subset Who Reported Receiving an mRNA formula of the Vaccine.
