# Supplementary material for: Emerging Socioeconomic Disparities in COVID-19 Vaccine Second-Dose Completion Rates in the United States
Source: Vaccines (Basel). 2022 Jan 14;10(1):121. doi: 10.3390/vaccines10010121 (PMC8780621; doi:10.3390/vaccines10010121)
Supplement: Supplementary file 1 [file vaccines-10-00121-s001.zip › vaccines-1500402-supplementary.pdf]

Supplementary Materials

Table S1. COVID-19 Vaccination Initiation and Completion Rates by Demographics, Stratified by Healthcare Worker Status.

	Healthcare Workers			Non-Healthcare Workers		
Demographic	Total [a] No. (%)	Dose Initiation [b] (95% MOE) [c]	Dose Completion [b] (95% MOE) [c]	Total [a] No. (%)	Dose Initiation [b] (95% MOE) [c]	Dose Completion [b] (95% MOE) [c]
Age (years)						
18–34	25.581 (17.9)	60.9 (60.1–61.7)	88.5 (87.7–89.3)	125,748 (17.9)	41.8 (41.4–42.2)	80.8 (80.4–81.2)
35–44	28.926 (20.3)	70.0 (69.3–70.7)	91.3 (90.5–92.1)	105,400 (15.0)	47.7 (47.3–48.1)	82.8 (82.4–83.2)
45–54	36.591 (25.6)	77.8 (77.2–78.4)	91.7 (91.1–92.3)	131,355 (18.7)	57.6 (57.2–58.0)	84.3 (84.0–84.6)
55–64	36.800 (25.8)	84.8 (84.3–85.3)	93.3 (92.8–93.8)	156,274 (22.3)	67.9 (67.6–68.2)	84.8 (84.5–85.1)
≥ 65	14.605 (10.2)	88.8 (88.1–89.5)	93.5 (92.7–94.3)	170,989 (24.4)	81.9 (81.7–82.1)	87.0 (86.8–87.2)
Missing	236 (0.20)	Suppressed [d]	Suppressed [d]	11,480 (1.6)	Suppressed [d]	Suppressed [d]
Race						
White	88,557 (62.0)	75.1 (74.7–7.5))	92.8 (92.5–93.1)	468,511 (66.8)	58.9 (58.7–59.1)	85.1 (84.9–85.3)
Black	22,961 (16.1)	63.1 (62.1–64.1)	86.7 (85.9–87.5)	92,304 (13.2)	48.5 (48.0–49.0)	80.8 (80.3–81.3)
Hispanic	14,421 (10.1)	70.3 (69.1–71.5)	89.0 (88.1–89.9)	72,684 (10.4)	51.2 (50.7–51.7)	80.9 (80.3–81.5)
Asian	9275 (6.5)	86.9 (86.7–87.0)	92.1 (91.2–93.0)	31,288 (4.5)	65,6 (64.8–66.4)	83.5 (82.8–84.2)
Other	7525 (5.3)	61.9 (60.2–63.6)	90.3 (89.0–91.6)	36,459 (5.2)	44.9 (44.1–45.7)	82.8 (82.0–83.6)
Essential Worker						
Yes	118,515 (83.0)	74.3 (73.9–74.7)	92.0 (91.6–92.4)	260,567(37.2)	53.3 (53.0–53.6)	84.2 (83.9–84.5)
No	23,876 (16.7)	68.4 (67.5–69.3)	88.4 (87.7–89.1)	176,508 (25.2)	60.5 (60.2–60.8)	84.3 (84.0–84.6)
Missing	347 (0.2)	65.1 (57.3–72.9)	88.7 (82.5–94.9)	264,171 (37.7)	51.9 (49.8–54.0)	86.2 (84.3–88.1)
Annual Household Income						
<30,000	18,189 (12.7)	55.7 (55.6–55.8)	84.9 (83.8–86.0)	138,166 (19.7)	43.0 (42.6–43.4)	79.8 (79.3–80.3)
30–49,999	20,601 (14.4)	67.1 (66.1–68.1)	88.6 (87.8–89.4)	96,677 (13.8)	53.8 (53.3–54.3)	82.8 (82.3–83.3)
50–74,999	23,330 (16.3)	73.9 (73.0–74.8)	91.2 (90.6–91.8)	110,009 (15.7)	58.3 (57.9–58.7)	84.2 (83.8–84.6)
75–99,999	20,585 (14.4)	77.5 (76.6–78.4)	92.7 (92.1–93.3)	89,608 (12.8)	61.4 (60.9–61.9)	85.0 (84.6–85.4)
100–150,000	26,218 (18.4)	81.2 (80.8–81.6)	93.6 (93.1–94.1)	110,663 (15.8)	64.4 (64.0–64.8)	85.6 (85.2–86.0)
>150,000	26,289 (18.5)	86.2 (85.6–86.8)	95.3 (94.9–95.7)	107,779 (15.4)	67.9 (67.5–68.3)	86.7 (86.3–87.1)

Missing	7427 (5.2)	75.8 (74.3–77.3)	92.2 (91.1–93.3)	48,344 (6.9)	62.8 (62.1–63.5)	85.1 (84.5–85.7)
Health Insurance						
Plan through employer	89,355 (62.6)	78.2 (77.8–78.6)	93.3 93.0–93.6)	310,601 (44.3)	59.2 (58.9–59.5)	84.7 (84.5–84.9)
Medicare	11,288 (7.9)	69.2 (67.9–70.5)	89.6 (88.6–90.6)	160,661 (22.9)	67.5 (67.2–67.8)	85.4 (85.1–85.7)
Self-purchased plan	18,366 (12.9)	72.2 (71.2–73.2)	90.2 (89.4–91.0)	73,414 (10.5)	56.1 (55.6–56.6)	84.2 (83.7–84.7)
Medicaid or Medi-Cal	6643 (4.7)	53.8 (51.9–55.7)	83.9 (81.1–86.8)	51,297 (7.3)	40.4 (39.8–41.0)	79.1 (78.3–79.9)
Tricare	2188 (1.5)	71.5 (68.6–74.5)	92.1 (90.1–94.1)	13,372 (1.9)	58.8 (57.5–60.1)	84.5 (83.4–85.6)
Other	7810 (5.5)	70.8 (69.2–72.4)	87.0 (85.7–88.4)	42,039 (6.0)	52.9 (52.2–53.6)	81.8 (81.1–82.5)
Uninsured	5800 (4.1)	52.0 (50.0–54.0)	83.5 (81.5–85.5)	40,170 (5.7)	33.5 (32.8–34.2)	77.0 (76.0–78.0)
Missing	1289 (0.9)	66.3 (62.3–70.3)	90.0 (87.0–93.0)	9692 (1.4)	47.4 (45.9–48.9)	82.1 (80.6–83.7)
Political Party						
Republican	33,182 (23.2)	68.5 (67.7–69.3)	91.9 (91.4–92.4)	155,519 (22.2)	52.2 (51.8–52.6)	84.4 (84.0–84.8)
Democrat	56,021 (39.2)	81.8 (81.3–82.3)	91.7 (91.3–92.1)	276,405 (39.4)	67.2 (67.0–67.4)	84.3 (84.1–84.5)
Independent	45,941 (32.2)	69.4 (68.7–70.1)	90.9 (90.4–91.4)	235,409 (33.6)	50.3 (50.0–50.6)	83.0 (82.7–83.3)
Missing	7595 (5.3)	72.4 (70.8–74.0)	90.6 (89.4–91.8)	33,913 (4.8)	53.9 (53.2–54.7)	83.2 (82.4–84.0)

[a] Crude, unweighted responses; [b] rates are weighted for gender, age, race, education, geography, profession, and political identification to match national general population; [c] margin of error (MOE); [d] sub-groups with insufficient numbers of responses were suppressed.

Table S2. Full Regression Output for Association between Annual Household Income and COVID-19 Vaccine Completion.

Demographic	AOR [a]	95% CI	*p*-Value
Annual Household Income			
<30,000	0.82	0.78–0.86	<0.001
30–49,999	0.92	0.88–0.96	<0.001
50–74,999	REF	--	--
75–99,999	1.07	1.03–1.11	<0.001
100–150,000	1.13	1.10–1.16	<0.001
>150,000	1.20	1.17–1.23	<0.001
Missing	1.06	1.01–1.11	0.05
Age (years)			
18–34	0.87	0.84–0.90	<0.001
35–44	0.95	0.92–0.98	0.01
45–54	REF	--	--
55–64	1.02	0.99–1.05	1.00
≥ 65	1.23	1.19–1.27	<0.001
Missing	0.08	-2.33–2.49	0.05
Race			
White	REF	--	--
Black	0.77	0.74–0.80	<0.001
Hispanic	0.93	0.90–0.96	<0.001
Asian	0.97	0.93–1.01	1.00
Other	0.90	0.85–0.95	<0.001
Essential Worker			
Yes	REF	--	--
No	1.13	1.11–1.15	<0.001
Missing	1.17	1.05–1.29	0.01
Industry			
Healthcare	REF	--	--
Education	0.48	0.45–0.51	<0.001
Government	0.57	0.53–0.61	<0.001
Other	0.50	0.47–0.53	<0.001
Missing	0.63	0.58–0.68	<0.001
Health Insurance			
Plan through employer	REF	--	--
Medicare	0.90	0.86–0.94	<0.001
Self-purchased plan	0.95	0.92–0.98	<0.001
Medicaid or Medi-Cal	0.82	0.77–0.87	<0.001
Tricare	1.04	0.97–1.11	1.00
Other	0.81	0.77–0.85	<0.001
Uninsured	0.71	0.66–0.76	<0.001
Missing	0.85	0.76–0.94	<0.001
Political Party			
Republican	0.89	0.86–0.92	<0.001
Democrat	REF	--	
Independent	0.92	0.90–0.94	<0.001
Missing	0.85	0.80–0.90	<0.001

[a] Adjusted odds ratio (AOR); adjusted for annual income, age, race, essential worker status, professional industry, health insurance coverage, and political party.

Table S3. Full Regression Output for Association between Annual Household Income and COVID-19 Vaccine Completion, Stratified by Healthcare Worker Status.

	Healthcare Worker			Non-Healthcare Worker		
Demographic	AOR [a]	95% CI	*p*-value	AOR[2]	95% CI	*p*-value
Annual Household Income						
<30,000	0.72	0.64–0.80	<0.001	0.86	0.82–0.90	<0.001
30–49,999	0.84	0.77–0.91	<0.001	0.95	0.91–0.99	0.01
50–74,999	REF	--	--	REF	--	--
75–99,999	1.10	1.02–1.18	0.05	1.06	1.02–1.10	0.01
100–150,000	1.29	1.21–1.27	<0.001	1.10	1.06–1.14	<0.001
>150,000	1.66	1.54–1.78	<0.001	1.20	1.16–1.24	<0.001
Missing	1.14	1.09–1.19	0.05	1.06	1.01–1.11	0.05
Age (years)						
18–34	0.85	0.78–0.92	<0.001	0.87	0.83–0.91	<0.001
35–44	1.02	0.95–1.09	1.0	0.93	0.90–0.96	<0.001
45–54	REF	--	--	REF	--	--
55–64	1.21	1.11–1.31	<0.001	0.99	0.95–1.03	1.00
≥65	1.56	1.46–1.66	<0.001	1.19	1.15–1.23	<0.001
Missing	--	--	--	0.08	-2.32–2.48	0.05
Race						
White	REF	-	--	REF	--	--
Black	0.60	0.53–0.63	<0.001	0.85	0.82–0.88	<0.001
Hispanic	0.83	0.75–0.90	<0.001	0.97	0.93–1.01	0.10
Asian	0.98	0.89–1.07	1.00	0.96	0.91–1.01	1.00
Other	0.74	0.64–0.84	<0.001	0.95	0.90–1.00	0.05
Essential Worker						
Yes	REF	--	--	REF	--	--
No	1.72	1.67–1.77	<0.001	1.06	1.04–1.08	<0.001
Missing	1.07	0.68–1.46	1.0	1.28	1.16–1.40	<0.001
Health Insurance						
Plan through employer	REF	--	--	REF	--	--
Medicare	0.66	0.56–0.76	<0.001	0.95	0.91–099	0.05
Self-purchased plan	0.81	0.74–0.88	<0.001	0.99	0.96–1.02	1.00
Medicaid or Medi-Cal	0.60	0.50–0.70	<0.001	0.90	0.84–0.96	<0.001
Tricare	0.90	0.72–1.08	1.0	1.10	1.02–1.18	0.05
Other	0.70	0.61–0.79	<0.001	0.85	0.82–0.88	<0.001
Uninsured	0.56	0.46–0.66	<0.001	0.74	0.69–0.79	<0.001
Missing	--	--	--	0.92	0.82–1.02	0.10
Political Party						
Republican	0.78	0.72–0.84		0.92	0.89–0.95	<0.001
Democrat	REF	--	--	REF	--	--
Independent	0.85	0.80–0.90		0.94	0.91–0.97	<0.001
Missing	0.79	0.69–0.89		0.89	0.84–0.94	<0.001

[a] Adjusted odds ratio (AOR); adjusted for annual income, age, race, essential worker status, health insurance coverage, and political party.

Table S4. COVID-19 Vaccination Initiation and Completion Rates by Demographics, Prior to Approval and Administration of Viral-Vector Vaccine [a].

Demographic	Dose Initiation [b] (95% MOE) [c]	Dose Completion [b] (95% MOE) [c]
Age (years)		

18–34	9.7(9.1–10.2)	64.9 (62.5–67.3)
35–44	12.8 (12.1–13.5)	62.1 (60.0–64.2)
45–54	15.9 (15.3–16.5)	62.6 (60.9–64.3)
55–64	20.1 (19.4–20.8)	56.1 (54.6–57.6)
≥65	52.6 (51.8–53.4)	47.7 (46.7–48.8)
Missing	Suppressed [d]	Suppressed [d]
Race		
White	23.1 (22.7–23.5)	54.1 (53.3–54.9)
Black	15.4 (14.5–16.3)	53.2 (50.7–55.7)
Hispanic	15.2 (14.2–16.2)	52.9 (50.0–55.8)
Asian	21.8 (20.1–23.5)	62.5 (59.3–65.7)
Other	15.6 (14.3–16.9)	56.9 (53.3–60.5)
Essential Worker		
Yes	19.9 (19.4–20.4)	65.1 (64.1–66.2)
No	16.0 (15.4–16.6)	46.6 (44.7–48.5)
Missing	17.4 (12.5–22.4)	58.0 (44.4–71.6)
Annual Household Income		
<30,000	11.5 (10.9–12.1)	53.1 (50.7–55.5)
30–49,999	18.3 (17.4–19.2)	48.9 (46.8–51.0)
50–74,999	22.1 (21.2–23.0)	53.5 (51.7–55.3)
75–99,999	24.4 (23.4–25.4)	52.7 (50.8–54.6)
100–150,000	26.5 (25.6–27.4)	56.8 (55.2–58.4)
>150,000	28.6 (27.6–29.6)	59.7 (8.1–61.3)
Missing	27.8 (26.3–29.3)	56.7 (54.2–59.2)
Health Insurance		
Plan through employer	18.6 (18.1–19.1)	59.9 (58.9–60.9)
Medicare	37.5 (36.6–38.4)	48.1 (46.9–49.4)
Self-purchased plan	17.8 (16.8–18.8)	57.8 (55.4–60.2)
Medicaid or Medi-Cal	8.1 (7.2–9.1)	51.9 (47.1–56.7)
Tricare	24.7 (21.9–27.5)	54.0 (48.6–59.4)
Other	18.3 (16.9–19.7)	52.8 (49.6–56.0)
Uninsured	6.1 (5.2–7.0)	60.1 (54.3–65.9)
Missing	Suppressed *	Suppressed *
Political Party		
Republican	21.1 (20.3–21.9)	57.2 (55.5–58.9)
Democrat	23.7 (23.1–24.3)	52.2 (51.0–53.4)
Independent	16.8 (16.2–17.4)	55.2 (53.7–56.7)
Missing	20.4 (18.8–22.0)	54.9 (51.2–58.6)

[a] Survey responses 8 February to 7 March 2021; [b] rates are weighted for gender, age, race, education, geography, profession, and political identification to match national general population; [c] margin of error (MOE); [d]sub-groups with insufficient numbers of responses were suppressed.

Table S5. COVID-19 Vaccination Completion Rates by Demographics, among Subset who Reported Receiving an mRNA formula of the Vaccine [a].

**Demographic**	**Dose Completion [b] (95% MOE) [c]**
Age (years)	
18–34	81.7 (81.3–82.1)
35–44	83.9 (83.5–84.3)
45–54	85.7 (85.4–86.0)
55–64	86.5 (86.2–86.8)
≥ 65	91.2 (91.0–91.4)

Missing	Suppressed [d]
Race	
White	87.2 (87.0–87.4)
Black	83.2 (82.7–83.7)
Hispanic	82.9 (82.4–83.4)
Asian	85.8 (85.1–86.5)
Other	85.3 (84.5–86.1)
Essential Worker	
Yes	86.3 (86.1–86.5)
No	85.4 (85.1–85.7)
Missing	87.5 (85.5–89.5)
Annual Household Income	
<30,000	81.4 (80.9–81.9)
30–49,999	84.7 (84.3–85.1)
50–74,999	86.3 (85.9–86.7)
75–99,999	87.1 (86.7–87.5)
100–150,000	87.8 (88.4–89.0)
>150,000	88.7 (88.4–89.0)
Missing	87.6 (87.1–88.1)
Health Insurance	
Plan through employer	86.4 (86.2–86.6)
Medicare	89.1 (88.8–89.4)
Self-purchased plan	85.6 (85.1–86.1)
Medicaid or Medi-Cal	79.6 (78.8–80.4)
Tricare	87.1 (86.0–88.2)
Other	83.9 (83.2–84.6)
Uninsured	78.3 (77.3–79.3)
Missing	83.6 (82.0–85.2)
Political Party	
Republican	86.4 (86.1–86.7)
Democrat	86.6 (86.4–86.8)
Independent	85.0 (84.7–85.3)
Missing	85.3 (84.6–86.0)

[a] Survey responses beginning 7 March 2021 and restricted to those who answered an additional question about type of vaccine with an mRNA formulation; [b] rates are weighted for gender, age, race, education, geography, profession, and political identification to match national general population; [c] margin of error (MOE); [d] sub-groups with insufficient numbers of responses were suppressed.
